# Liver metastases of neuroendocrine tumours; early reduction of tumour load to improve life expectancy

**DOI:** 10.1186/1477-7819-4-35

**Published:** 2006-06-26

**Authors:** Liesbeth M Veenendaal, Inne HM Borel Rinkes, Cornelis JM Lips, Richard van Hillegersberg

**Affiliations:** 1 Department of Surgery, University Medical Center Utrecht, The Netherlands; 2 Department of Clinical Endocrinology, University Medical Center Utrecht, The Netherlands

## Abstract

**Background:**

Neuroendocrine tumours frequently metastasize to the liver. Although generally slowly progressing, hepatic metastases are the major cause of carcinoid syndrome and ultimately lead to liver dysfunction, cardiac insufficiency and finally death.

**Methods:**

A literature review was performed to define the optimal treatment strategy and work-up in patients with neuroendocrine hepatic metastases. Based on this, an algorithm for the management of these patients was established.

**Results:**

Platelet serotonin and chromogranin A are useful biomarkers for detection and follow-up of neuroendocrine tumour. Helical computed tomography and somatostatin receptor scintigraphy are the most sensitive diagnostic modalities. Surgical debulking is an accepted approach for reducing hormonal symptoms and to establish better conditions for medical treatment, but is frequently impossible due to the extent of disease. A novel approach is the local ablation of tumour by thermal coagulation using therapies such as radiofrequency ablation (RFA) or laser induced thermotherapy (LITT). These techniques preserve normal liver tissue. There is a tendency to destroy metastases early in the course of disease, thereby postponing or eliminating the surgically untreatable stage. This can be combined with postoperative radioactive octreotide to eliminate small multiple metastases. In patients with extensive metastases who are not suitable for local destruction, systemic therapy by octreotide, ^131^I-MIBG treatment or targeted chemo- and radiotherapy should be attempted. A final option for selective patients is orthotopic liver transplantation.

**Conclusion:**

Treatment for patients with neuroendocrine hepatic metastases must be tailored for each individual patient. When local ablative therapies are used early in the course of the disease, the occurrence of carcinoid syndrome with end stage hepatic disease can be postponed or prevented.

## Background

Carcinoids are neuroendocrine tumours that arise from neoplastic proliferation of enterochromaffin or Kulchitsky cells [1]. In 1963, carcinoids were classified according to their embryologic site of origin as foregut carcinoids (respiratory tract, stomach, duodenum, biliary system and pancreas), midgut carcinoids (small intestine, appendix, cecum, and proximal colon), and hindgut carcinoids (distal colon and rectum) [2]. According to the WHO classification in 2000, distinction was made between well-differentiated neuroendocrine tumours (benign behaviour or uncertain malignant potential, <2% Ki67 positive cells), well-differentiated neuroendocrine carcinomas (low grade malignancy, presence of metastasis and/or invasiveness), and poorly differentiated neuroendocrine carcinomas of high-grade malignancy (usually small cell, >15% Ki67 positive cells) [3]. Ki67 is an immunohistochemical biomarker for cell proliferation.

Neuroendocrine hepatic metastases represent about 10% of all hepatic metastatic neoplasm's [4]. These metastases occur in about 25–90% of patients with neuroendocrine tumours. Although these tumours run a rather indolent course, the 5-year survival of patients with neuroendocrine tumours and liver metastases is 40% compared with 75–99% in those free of liver metastases [5-7]. Neuroendocrine liver metastases often progress slowly but may cause significant symptoms due to their size and/or hormone production. Ultimately the hepatic tissue is replaced by tumour, causing mechanical pressure to surrounding tissues, liver dysfunction, cardiac failure and finally death. Manifestations of the carcinoid syndrome usually occur in patients with liver metastases due to production and release of serotonin directly in the blood stream. Classically, the carcinoid syndrome is characterised by episodic flushing, tachycardia, diarrhoea and bronchospasm [8]. Treatment of neuroendocrine hepatic metastases is aiming at symptomatic improvement and reduction of hormonal hypersecretion by elimination of the tumour. However, the most effective management and timing of treatment remains unclear [9,10]. Here, we have reviewed the literature and used our own experience to provide a balanced guideline for imaging and management of patients with neuroendocrine hepatic metastases.

## Biochemical diagnosis

Neuroendocrine tumours of the small intestine produce large quantities of serotonin (5-hydroxytryptamine), reflected in raised levels of platelet serotonin and a high urinary excretion of 5-hydroxyindoleacetic acid (5-HIAA) [11,12]. The platelet serotonin concentration is more sensitive in the detection of carcinoid tumours than urinary 5-HIAA, particularly in tumours with relatively low serotonin production [13,14]. Circulating free serotonin is removed very rapidly and effectively by the liver. In contrast to urinary 5-HIAA, platelet serotonin is not effected by serotonin-containing diet [15]. Hence platelet serotonin is the most discriminating marker for detection of most neuroendocrine tumours. However, in hindgut carcinoids, hydroxylase and decarboxylase are absent and no serotonin is produced.

Plasma chromogranin A (CgA) has been claimed the most sensitive and specific marker of tumour volume [16]. CgA is a precursor for several peptides and is stored in secretory granules of neuroendocrine tissue [17]. Circulating CgA allows early detection of persistent or recurrent neuroendocrine tumours [18]. The highest CgA levels were noted in metastatic midgut lesions [19].

Both tumour markers, platelet serotonin and CgA, can be reliably used for diagnosis of neuroendocrine tumour and for monitoring the outcome of treatment in individual patients.

## Work-up of patients with neuroendocrine hepatic metastases

Several imaging modalities are available to detect hepatic metastases and their primary neuroendocrine tumours. Conventional ultrasonography (US), computed tomography (CT), magnetic resonance imaging (MRI) and somatostatin receptor scintigraphy (SRS) are the cornerstones for the localisation of neuroendocrine tumours with sensitivities of respectively 46%, 42%, 43% and 90% [20-23]. The use of helical computed tomography (hCT) has increased the diagnostic sensitivity in the localisation of both primary (94%) and metastatic tumour (lymph node 69%, liver 94%) [24]. As somatostatin receptor subtype 2 is present in almost 80% of neuroendocrine tumours, binding 111Indium-labelled octreotide can be used for both disease staging and to indicate whether or not somatostatin analogues can be used in the treatment of these tumours [25]. SRS is very helpful in detecting bone and lung metastases and thereby aids in confirming or refuting the presence of extrahepatic disease. Based on these considerations, both hCT and SRS should be performed in all patients prior to treatment.

## Treatment modalities

### Surgical resection

Surgical resection is to be considered when no extrahepatic disease is present. Hemihepatectomy or segmental resection is feasible when metastases are solitary and resection can be radical with enough functional liver tissue remaining. Symptomatic response rates have been reported to be 90% for a mean duration of 19.3 months after surgical resection [26]. Unfortunately, neuroendocrine metastases are usually multiple and diffuse and therefore resection is often impossible. Furthermore, in most patients treated by surgical resection with curative intent, additional metastases develop that presumably were occult at the time of surgery [26]. Therefore even in resectable cases, liver tissue sparing therapies should be considered, allowing future repeated treatment.

### Local ablative therapy

**Figure 1:**
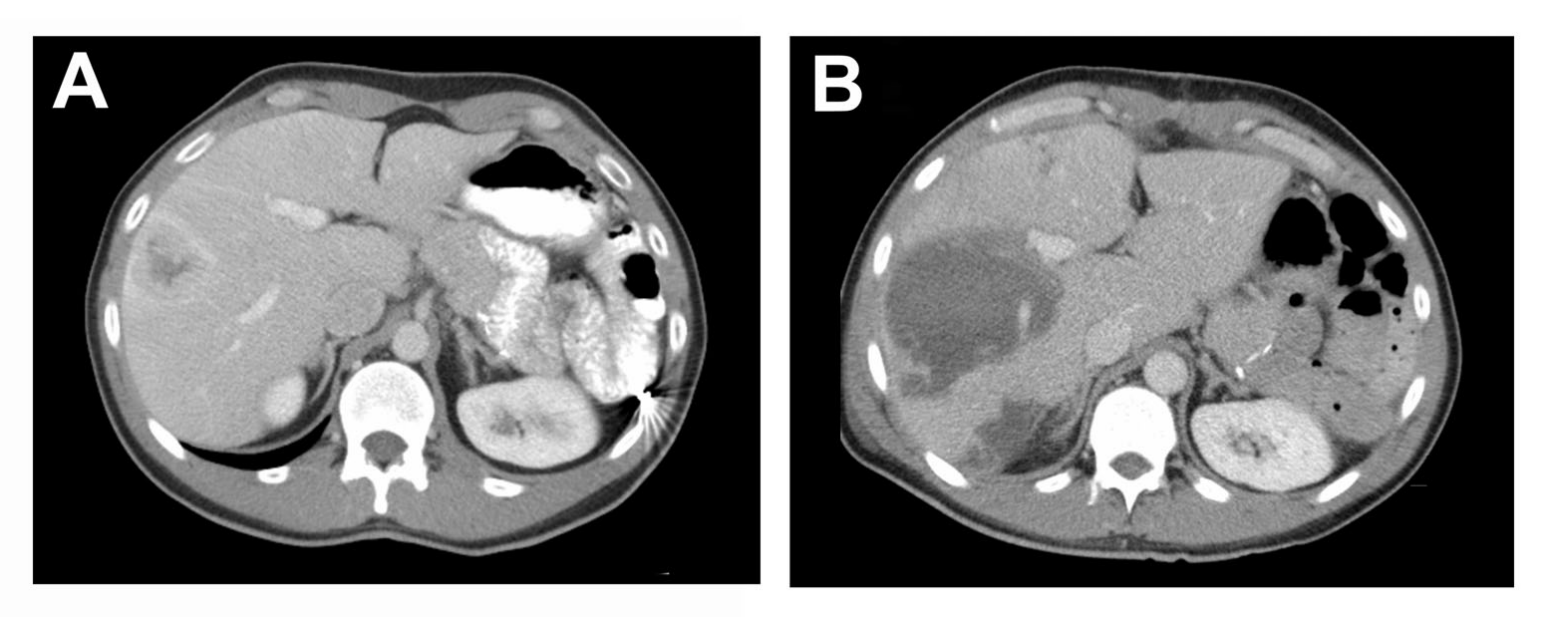
CT scan of the liver of a 34-year old man with metastases of a neuroendocrine tumour of the pancreas. Before LITT the CT scan shows a metastases of 4.7 cm in diameter in segment VII and a second metastases of 2.0 cm in diameter in segment VII subcapsular (not visible) of the liver (figure 2A). Control CT scan one week after LITT showing a coagulation lesion in segment VII of 9.0 cm in diameter and subcapsular in segment VII of 4.8 cm in diameter (figure 2B).

Local therapy using radiofrequency ablation (RFA) or laser induced thermotherapy (LITT) is a well-established treatment for unresectable hepatocellular carcinomas and liver metastases from colorectal carcinomas [27,28]. A few small series and case reports have also shown good response in neuroendocrine hepatic metastases [29-33]. Up until now, a disadvantage of these therapies has been the relatively small volume of tissue that can be coagulated. Clinical trials with RFA have shown that complete tumour eradication is more likely to occur with small tumours, i.e. diameter ≤ 4 cm, than with large tumours [34]. With the use of simultaneous multiple fiber LITT or next generation bipolar RFA, we have been able to ablate tumours as large as 7 cm in diameter [35] (Figure 1). Furthermore, up to 7 lesions at one time may be ablated using specialized techniques to increase lesion size [36]. It has been reported that cytoreduction of ≥ 90% is adequate for durable symptomatic relief [9]. In our most recent strategy, we aim at complete destruction of the intrahepatic tumour to prevent the occurrence of surgically untreatable disease. The largest reported study of 34 patients with neuroendocrine hepatic metastases treated with RFA showed symptom relief in 95% of these patients with significant or complete symptom control in 80%, for a mean of 10 months [29]. Even in patients with extrahepatic disease and liver metastases ablation may also provide symptom relief [29]. The complication rate is 5–10% and the mortality rate is about 0.5% [37-39]. Therefore these techniques are especially suitable for repeated treatment in patients in which local recurrence or new metastases developing during follow up.

All invasive procedures during surgery such as liver resection and ablation and even anaesthesia can induce hormone release and even provoke a life-threatening carcinoid crisis [40]. In the severe crisis of carcinoid syndrome the flush is usually accompanied by hypotension and occasionally shock. Injection of octreotide, the long-acting analog of somatostatin, usually prevents or aborts this vasomotor reaction [41]. Studies have shown that the use of octreotide intraoperatively for patients with metastatic carcinoid tumours undergoing surgery with manipulation of tumour is associated with a decreased frequency of intraoperative complications [42,43].

### Arterial embolisation

Hepatic arterial embolisation with or without chemotherapy is a palliative option for those unresectable lesions in which the predominant mass of tumour is localised in one of the liver lobes. In the past, more radical blunt techniques to occlude the main hepatic artery were used. However, recently, superselective techniques have become available with the advantage of leaving the main segmental arteries open. Contraindications of hepatic arterial embolisation include complete portal vein occlusion, hepatic failure and previous biliary anastomoses [44]. Symptomatic improvement after hepatic arterial embolisation is reported to occur in 64–90% [45,46]. Reports on chemoembolisation show a slight better biochemical response and tumour response than hepatic artery embolisation [47]. Embolisation techniques are associated with mortality rates of about 5% and almost all patients develop the 'postembolic syndrome' (elevated liver function tests and fever) although mostly transient and in different grades of severity [48-50]. In addition, serious complications have occurred in about 10% of patients treated with hepatic embolisation for neuroendocrine tumours [51]. Complications can be reduced by prophylactic octreotide infusion during the procedure and the use of forced diuresis during and after the embolisation. In case of partial or no response, supplementary embolisation or additional RFA or LITT could be an option. In selected cases with good response to embolisation a partial hepatic resection may be considered.

### Pharmacological therapy

Pharmacological therapy consists of long-lasting octreotide injections, Iodine-131 metaiodobenzylguanidine (^131^I-MIBG), interferon-α (IFN-α) or targeted chemo- and radiotherapy. Octreotide is a somatostatin analogue and appears to be an efficacious treatment for carcinoid syndrome, reducing symptoms in more than 70% of patients [52,53]. Some patients with partial response after local ablation have relief of symptoms by additional treatment with octreotide [37]. Prolonged symptomatic relief can be provided by ^131^I-MIBG therapy. In individual cases, improved quality of life may be obtained [54]. Even improved survival was seen by symptomatic response to ^131^I-MIBG treatment [55]. The clinical benefit of IFN-α treatment has been limited by their modest anti-tumour effect as well as serious side-effects [56,57]. In addition, combination treatment with octreotide and IFN-α showed little advantage. Biochemical responses were observed in 72–77%, however no objective tumour regression was observed [57,58]. A promising approach is the concept of somatostatin receptor (SSTR)-mediated chemo-or radiotherapy of SSTR-expressing metastatic carcinoid. Currently, clinical trials with cytotoxic compounds, such as methotrexate and doxorubicin, linked to an analog of somotostatin are under way [59,60]. Also promising is targeted SSRT-mediated radiotherapy using radionuclides such as ^90^Y and ^177^Lu. Experimental studies in patients who have somatostatin-positive tumours show complete remission by the use of tetra-azacyclododecane tetra-acetic acid Tyr^3^-octreotide [61]. After surgical reduction of tumour load, repeated intermediate-dosage ^90^Y, Tyr-octreotide, ^177^Lu or ^131^I-MIBG treatment appears to be a reliable and well-tolerated radionuclide therapy and might be a useful adjunct in patients with malignant neuroendocrine carcinoma, providing long-lasting palliation and prolonged survival [62].

### Liver transplantation

Young patients with surgically unresectable tumours, hepatomegaly and uncontrollable symptoms, in whom all other therapies have been unsuccessful, may benefit from liver transplantation [63]. However, liver transplantation for metastatic disease is controversial and in most cases even contraindicated, as the results have been poor due to complex operative procedures [64-66]. Well differentiated tumours and a low proliferation rate (Ki67<10%) are important selection criteria [67]. Overall, post-operative mortality of 19% is reported in a group of 31 patients undergoing orthotopic liver transplantation for metastatic neuroendocrine tumours [65]. In the same study, 50% of the carcinoid patients suffered from one or more major complications i.e. peritoneal bleeding, acute/chronic rejection and acute pancreatitis [65].

## Conclusion

**Figure 2:**
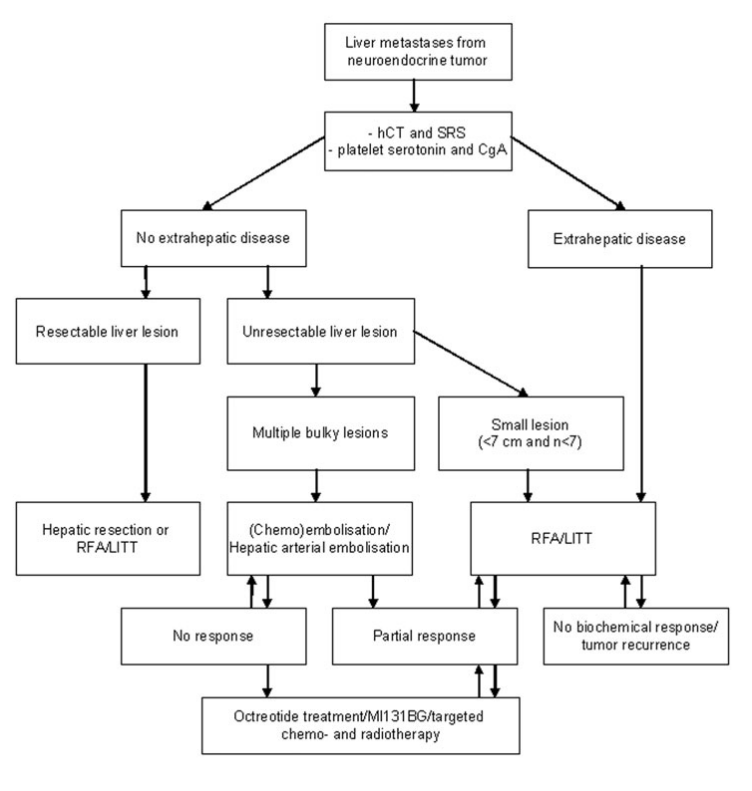
Protocol for management of patients with neuroendocrine hepatic metastases. CT, computed tomography; MRI, magnetic resonance imaging; SRS, somatostatin receptor scintigraphy; RFA, radiofrequency ablation; LITT, laser induced thermotherapy; ^131^I-MIBG, Iodine-131 metaiodobenzylguanidine.

Hepatic metastases are frequently encountered in patients with digestive endocrine tumours and their presence plays an important role in quality of life and overall prognosis. Tailored multimodality treatment is the key to increase survival and achieve good palliation in patients with hepatic metastases from neuroendocrine tumours. A flow sheet such as presented in figure 2 can be helpful in the decision of choice of treatment. Determination of platelet serotonin and plasma CgA is useful for detection of neuroendocrine tumour and to evaluate therapy efficiency. Visualisation of neuroendocrine hepatic metastases should be performed by hCT/MRI and SRS. Determination of platelet serotonin and plasma CgA is useful for detection of neuroendocrine tumour and to evaluate therapy efficiency. The proliferation marker Ki67 is a very important tool in guiding the type of treatment. Surgery is the treatment of choice for hepatic metastases however cure is frequently impossible due to the extent of disease. Treatment aimed at cytoreduction of hepatic metastasis and diminished secretion of bioactive amines may achieve good palliation. Tumour destruction by RFA or LITT provides a novel liver preserving option. These techniques will now be used more often as liver preserving option to treat patients early in the course of their disease postponing drug intervention and preventing the end stage carcinoid syndrome and thereby improving life expectancy.

## Competing interests

The author(s) declare that they have no competing interests.

## Authors' contributions

**LV **reviewed the literature and drafted the manuscript. **IBR **and **CL **critically reviewed the paper and were involved in the preparation of the final manuscript. **RH **was involved in the conception of the work and manuscript preparation. All authors read and approved final version for publication.
